# Pulmonary vascular volume, impaired left ventricular filling and dyspnea: The MESA Lung Study

**DOI:** 10.1371/journal.pone.0176180

**Published:** 2017-04-20

**Authors:** Carrie P. Aaron, Eric A. Hoffman, Joao A. C. Lima, Steven M. Kawut, Alain G. Bertoni, Jens Vogel-Claussen, Mohammadali Habibi, Katja Hueper, David R. Jacobs, Ravi Kalhan, Erin D. Michos, Wendy S. Post, Martin R. Prince, Benjamin M. Smith, Bharath Ambale-Venkatesh, Chia-Ying Liu, Filip Zemrak, Karol E. Watson, Matthew Budoff, David A. Bluemke, R. Graham Barr

**Affiliations:** 1 Department of Medicine, College of Physicians and Surgeons, Columbia University, New York, NY, United States of America; 2 Department of Radiology, University of Iowa, Iowa City, IA, United States of America; 3 Department of Medicine, Johns Hopkins University School of Medicine, Baltimore, MD, United States of America; 4 Departments of Medicine and Epidemiology, Perelman School of Medicine at the University of Pennsylvania, Philadelphia, PA, United States of America; 5 Departments of Medicine and Epidemiology and Prevention, Wake Forest University School of Medicine, Winston Salem, NC, United States of America; 6 Department of Radiology, Johns Hopkins University School of Medicine, Baltimore, MD, United States of America; 7 Department of Radiology, Hannover Medical School, Hannover, Germany; 8 Division of Epidemiology and Community Health, University of Minnesota School of Public Health, Minneapolis, MN, United States of America; 9 Asthma and COPD Program, Northwestern University Feinberg School of Medicine, Chicago, IL, United States of America; 10 Department of Epidemiology, Johns Hopkins Bloomberg School of Public Health, Baltimore, MD, United States of America; 11 Department of Radiology, College of Physicians and Surgeons, Columbia University, New York, NY, United States of America; 12 Department of Medicine, McGill University Health Center, Montreal, QC, Canada; 13 Radiology and Imaging Sciences, National Institutes of Health/Clinical Center; Bethesda, MD, United States of America; 14 Department of Medicine, University of California, Los Angeles, CA, United States of America; 15 Department of Epidemiology, Mailman School of Public Health, Columbia University, New York, NY, United States of America; Cincinnati Children's Hospital Medical Center, UNITED STATES

## Abstract

**Background:**

Evaluation of impaired left ventricular (LV) filling has focused on intrinsic causes of LV dysfunction; however, pulmonary vascular changes may contribute to reduced LV filling and dyspnea. We hypothesized that lower total pulmonary vascular volume (TPVV) on computed tomography (CT) would be associated with dyspnea and decrements in LV end-diastolic volume, particularly among ever-smokers.

**Methods:**

The Multi-Ethnic Study of Atherosclerosis recruited adults without clinical cardiovascular disease in 2000–02. In 2010–12, TPVV was ascertained as the volume of arteries and veins in the lungs detectable on non-contrast chest CT (vessels ≥1 mm diameter). Cardiac measures were assessed by magnetic resonance imaging (MRI). Dyspnea was self-reported.

**Results:**

Of 2303 participants, 53% had ever smoked cigarettes. Among ever-smokers, a lower TPVV was associated with a lower LV end-diastolic volume (6.9 mL per SD TPVV), stroke volume, and cardiac output and with dyspnea (all P-values <0.001). Findings were similar among those without lung disease and those with 0–10 pack-years but were mostly non-significant among never-smokers. TPVV was associated smaller left atrial volume but not with LV ejection fraction or MRI measures of impaired LV relaxation. In a second sample of ever-smokers, a lower pulmonary microvascular blood volume on contrast-enhanced MRI was also associated with a lower LV end-diastolic volume (P-value = 0.008).

**Conclusion:**

Reductions in pulmonary vascular volume were associated with lower LV filling and dyspnea among ever-smokers, including those without lung disease, suggesting that smoking-related pulmonary vascular changes may contribute to symptoms and impair cardiac filling and function without evidence of impaired LV relaxation.

## Introduction

Heart disease and chronic obstructive pulmonary disease (COPD) are the first and third leading causes of morbidity and mortality in the world [1, 2]. Heart failure with preserved ejection fraction (HFpEF) is an increasingly common diagnosis with significant clinical implications and few therapies [3]. HFpEF and COPD both contribute to dyspnea and are frequently diagnosed in the same patient [4].

Evaluation of impaired left ventricular (LV) filling, a cardinal feature of HFpEF, has focused on intrinsic causes of LV dysfunction [5]. Given the high cross-sectional area and flow in the pulmonary circulation, minor but diffuse pulmonary vascular damage may decrease LV filling and impair gas exchange, contributing to dyspnea and a potential misdiagnosis of HFpEF. Alternatively, intrinsic impairment of LV relaxation would be expected to result in larger pulmonary vascular volumes. However, whether subclinical pulmonary vascular differences are associated with impaired LV filling in the general population is unknown.

Pulmonary vascular changes occur in emphysema, as it is defined by destruction of alveolar walls that include pulmonary capillaries [6]. We previously demonstrated in the general population that the percentage of emphysema-like lung (percent emphysema) on CT was associated with decreased LV filling [7] and dyspnea [8]. Lower lung function has also been associated with reduced LV filling and the development of heart failure [9]. A smaller study of smokers, mostly with COPD, showed that the loss of small pulmonary vessels on CT was associated with greater percent emphysema and reduced six minute walk distance [10]. Whether pulmonary vascular changes are relevant outside of COPD and emphysema, however, is unclear.

We therefore performed full-lung CTs in a population-based cohort to test whether lower total pulmonary vascular volume (TPVV) on CT is associated with impaired LV filling on MRI and dyspnea. We hypothesized *a priori* that associations would be of greater magnitude among ever-smokers. We also examined left atrial volume and MRI signs of LV relaxation. Finally, we tested whether a contrast-enhanced MRI measure of pulmonary microvascular blood volume was associated with lower LV filling in a second sample.

## Materials and methods

### Multi-Ethnic Study of Atherosclerosis

The Multi-Ethnic Study of Atherosclerosis (MESA) is a prospective cohort study that recruited 6814 participants from six U.S. communities who were white, African-American, Hispanic or Chinese-American and ages 45–84 years in 2000–02 and excluded those with clinical cardiovascular disease (angina, stroke or TIA, heart failure, atrial fibrillation, cardiac procedure), undergoing treatment for cancer, pregnancy, weight over 300 pounds, a chest CT scan in the prior year, cognitive inability or language barrier (other than English, Spanish, Cantonese or Mandarin), and conditions impeding long term follow up (serious medical condition, living in a nursing home, or plans to leave the community within five years) [11]. In 2010–12 4716 participants returned for follow-up. All were invited to undergo cardiac MRI at baseline; the 75% who did were invited to repeat it in 2010–12.

The MESA Lung Study enrolled 3965 MESA participants sampled randomly among those with baseline measures of endothelial function, consent for genetic analyses and attended an examination during the recruitment period in 2004–06 [12], and a random sample in 2010–12 undergoing cardiac MRI (S1 Fig); all were invited to undergo full-lung CT and spirometry in 2010–12.

The protocols of MESA and all studies described herein were approved by the Institutional Review Boards of all collaborating institutions (Columbia University Medical Center Institutional Review Board, the Johns Hopkins University School of Medicine Joint Committee on Clinical Investigation, the University of Minnesota Human Research Protection Program, the Northwestern University Social and Behavioral Sciences Institutional Review Board, the Harbor-University of California Los Angeles (UCLA) Research and Education Institute Human Subjects Committee, the UCLA Office of Human Research Protection Program, the University of Vermont Committees on Human Research, the Wake Forest University Health Sciences Office of Research Institutional Review Board, and the University of Washington Human Subjects Division) and the NHLBI. All participants provided written informed consent.

### Total pulmonary vascular volume on CT

Non-contrast chest CTs were acquired at suspended full inspiration on 64-detector scanners following the SPIROMICS protocol with reconstruction in 0.625–0.75mm increments by a high-spatial contrast algorithm [13]. Trained readers used dedicated software (Apollo, VIDA Diagnostics) to segment the lungs and pulmonary vessels with visual confirmation.

**Fig 1:**
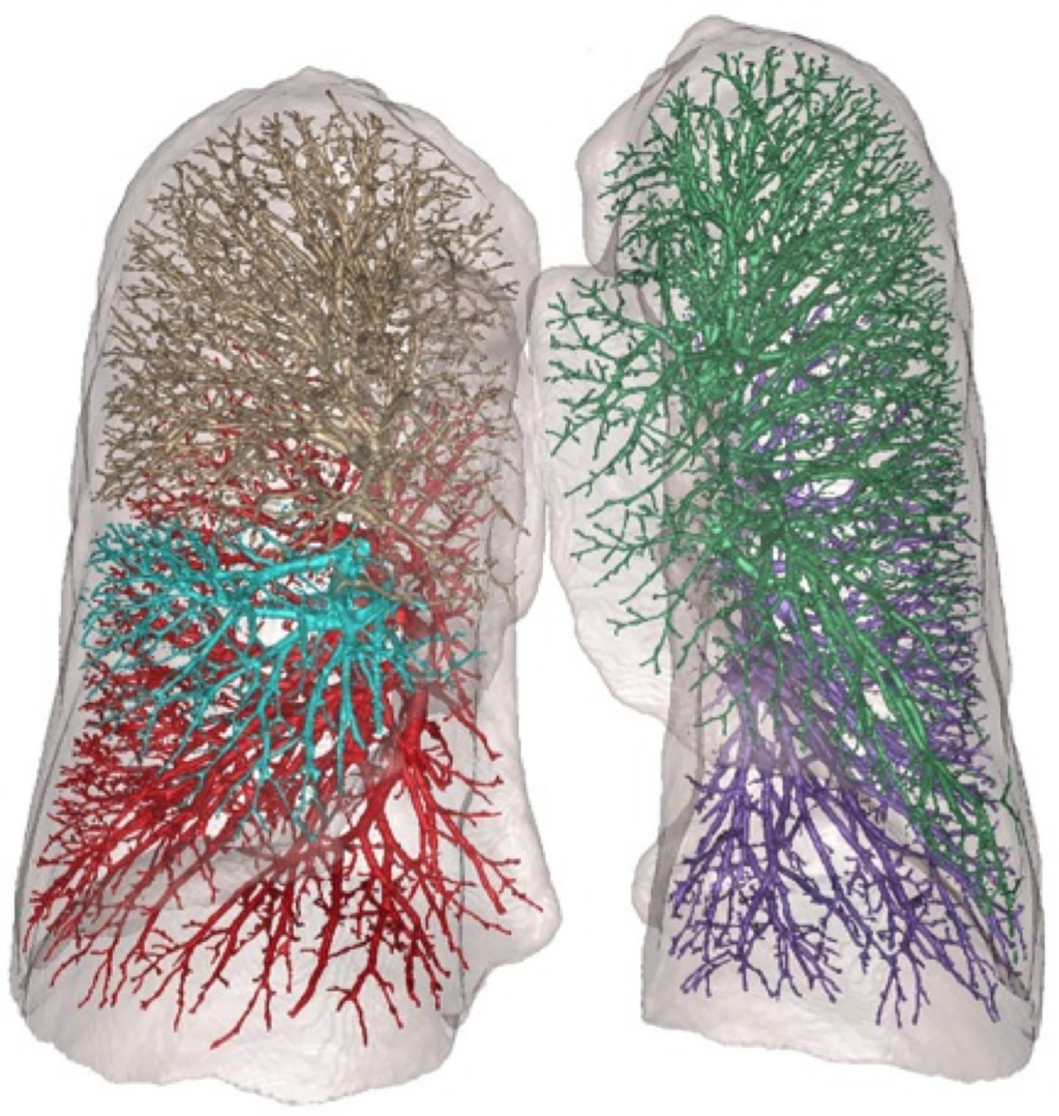
3-dimensional reconstruction of the vessels comprising the total pulmonary vascular volume and the lungs on CT scan. Colors depict the 5 lobes of the lungs; shaded region, the lung volume.

TPVV was measured within the segmented lung as the volume of arteries and veins, including vessel walls and luminal blood, down to approximately 1 mm in diameter (Fig 1) [14]. The intraclass correlation coefficient on 10% replicate reading was 1.0. Percent TPVV is TPVV indexed to CT lung volume. The TPVV (mean of 130.7 cm^3^ or 70.7 cm^3^/m^2^ in this study) captures approximately 25–30% of the pulmonary blood volume estimated using invasive methods (250–300 cm^3^/m^2^) [15, 16]. This difference is not unexpected, given that the TPVV does not capture the microvasculature (capillary blood volume, estimated as ~140 mL) [17], pre-capillary arterioles and venules, and that the main, right and left pulmonary arteries and veins were excluded from the TPVV but included in invasive measures.

### Cardiac magnetic resonance imaging

MRI imaging was performed on 1.5T scanners with a phased-array surface coil [18]. Analysts blinded to participant information assessed LV mass, volumes and function using cine steady-state free precession pulse sequence acquired on 2-chamber, 3-chamber, 4-chamber and short-axis planes [19]. LV mass, end-diastolic and end-systolic volume measurements used semiautomatic contouring (CIM6.0, UniServices) [20]. LV mass was determined at end-diastole as epicardial minus endocardial volume times myocardial specific gravity [19]. Stroke volume was calculated as end-diastolic volume minus end-systolic volume, LV ejection fraction as stroke volume divided by end-diastolic volume, and cardiac output as stroke volume times heart rate. The interobserver intraclass correlation coefficients were 0.96 for end-diastolic volume, and for 0.95 for mass.

Left atrial volumes were measured using CVI42 software (Circle Cardiovascular Imaging Inc.). Horizontal and vertical long-axis cine steady-state free precession images were used to measure the biplanar left atrial volume in end-systole [(8 x vertical area x horizontal area)/(3π x ((vertical length + horizontal length)/2))], excluding the pulmonary veins and left atrial appendage. The interobserver intraclass correlation coefficient for left atrial volume was 0.96. T_1_ mapping was performed on a subset to detect myocardial fibrosis [18].

Those eligible for gadolinium received an intravenous bolus of 0.15 mmol/kg (Magnevist, Bayer Healthcare Pharmaceuticals) and diffuse fibrosis was evaluated before and after contrast injection at 12 and 25 minutes on one short axis mid-slice using a modified Look-Locker inversion recovery (MOLLI) sequence over a single breath-hold. The inversion recovery echo triggered sequence was three inversion pulses at 100, 200 and 350 ms, and T_1_ mapping was performed using MASS research software (Department of Radiology, Leiden University Medical Center). The partition coefficient was calculated as the slope using 3 times points (ΔR_1myocardium_/ΔR_1blood_), and the extracellular volume (ECV) as (partition coefficient x 100 x [1 –hematocrit]) [21]. Greater myocardial fibrosis by MRI is indicated by lower post-contrast T_1_ time and higher ECV [22, 23].

LV circumferential diastolic strain (Δ length/mean length, %) was measured using the harmonic phase method on four mid-ventricular segments from short-axis-tagged slices [24]. Peak early diastolic strain rate was measured on strain-by-time curves as the peak rate of change in early diastolic strain (%/msec). The strain relaxation index is the ratio of early/total relaxation time divided by the peak early diastolic strain rate (msec/%) [25]. Lower peak early diastolic strain rate and higher strain relaxation index indicate impaired myocardial relaxation.

In the second sample with measures of pulmonary microvascular blood volume on contrast-enhanced MRI, left atrial volumes were measured using multimodality tissue-tracking software version 6.0 (Toshiba) [26]. RV parameters were measured using QMASS software version 4.2 (Medis) [27]. Ostial pulmonary vein cross-sectional area was assessed using multiplanar reformation software (Volume Viewer 15.10.4; General Electric) [28].

### Pulmonary microvascular blood volume on MRI

Pulmonary microvascular blood volume was measured on contrast-enhanced MRI among 142 participants who were ages 50–79 years and had smoked at least 10 pack-years [29]. Pulmonary microvascular blood flow and mean transit time were calculated from signal intensity-by-time curves following a 0.1 mmol/kg gadolinium bolus on 3D-spoiled gradient-recalled echo images at functional residual capacity. Pulmonary microvascular blood volume was calculated as blood flow times mean transit time [29]. Measurements were obtained in the peripheral 2cm on 1cm coronal slices. The mean pulmonary microvascular blood volume applied to the entire lung (142cm^3^ [76.6cm^3^/m^2^]) was similar to Weibel’s morphological estimate of 140mL [17].

### Respiratory symptoms

Trained interviewers assessed respiratory symptoms using standard questionnaires [30]. Dyspnea was defined as more breathlessness walking on level ground, hills or stairs compared to those of same age, or breathlessness that causes one to stop walking, i.e., a modified Medical Research Council score of 2 or greater [31]. Wheezing was defined as any wheeze within 12 months, and chronic cough as occurring for at least 3 of the past 12 months.

### Covariate information

Age, sex, race/ethnicity, education and medical history were self-reported. Ever-smoking was defined as over 100 lifetime cigarettes, current smoking as self-report of smoking within 30 days or urinary cotinine over 568 nmol/L [12], and pack-years as years smoking times packs/day. Height, weight, blood pressure, fasting glucose, serum creatinine, total cholesterol, high-density lipoprotein cholesterol (HDL-C) and triglycerides were measured using standard techniques [32]. Hypertension was defined as blood pressure ≥140/90 mmHg, or self-report and antihypertensive medication use. Diabetes was defined as fasting glucose ≥7.0 mmol/L or hypoglycemic medication use. Resting arterial hemoglobin saturation was measured by pulse oximetry (CMS-50F, Contec Medical Systems). Total body water was estimated by body composition scale (BCS-2, Valhalla Scientific). Physical activity was self-reported [33]. Agatston coronary artery calcium score was calculated on gated cardiac CTs [34]. Medication inventory assessed medication use [35].

Spirometry was conducted according to American Thoracic Society-European Respiratory Society guidelines [36], using National Health and Nutrition Examination Survey III equations for predicted values (0.88 correction-factor for Chinese-Americans) [37]. Airflow limitation was defined as pre-bronchodilator FEV_1_/FVC <0.7, and restrictive ventilatory defect as FVC <lower limit of normal and FEV_1_/FVC >0.7. Percent emphysema was defined as the percentage of voxels below -950 Hounsfield units (HU) on full-lung CTs (Vida Diagnostics). Reference equations defined emphysema on CT (percent emphysema above the upper limit of normal) and predicted total lung volume on CT [38]. Lung regions between -600 and -250 HU were considered high attenuation areas on CT, reflecting subclinical interstitial lung disease [39].

### Statistical analysis

Associations between TPVV and LV measures were estimated using generalized linear and additive regression. Preliminary models adjusted for age, sex, race/ethnicity, height, weight, education, CT manufacturer and milliamperes. Full models added smoking status, pack-years, cardiac risk factors, creatinine, diuretic use, lung function and percent emphysema. Additive interactions were tested in the full model for smoking history (ever vs. never), age, sex and race/ethnicity. Missing data was minimal except for spirometry (11%) and pack-years (6%), and was addressed by multiple imputation.

Associations between TPVV and respiratory symptoms were estimated using logistic regression. Preliminary models adjusted for age, sex, race/ethnicity, height, weight, smoking status, pack-years, CT manufacturer and milliamperes. Full models also included predictors of dyspnea: lung function, percent emphysema and LV ejection fraction.

Statistical significance was defined by a two-tailed P-value <0.05. Analyses were performed using SAS 9.3 (SAS Institute) and R package 3.2.3 (The R Project).

## Results

### Study participants

Of 4716 participants evaluated in 2010–12, 3136 underwent full-lung CT, of whom 2303 completed cardiac MRI and were included in analyses (S1 Fig). There were small differences in demographics, smoking, percent emphysema and TPVV between included and excluded participants (S1 Table).

Participants were a mean (±SD) age of 69±9 years (range 54–94), 49% male, 40% white, 27% African-American, 20% Hispanic and 14% Chinese-American. Fifty-three percent reported ever smoking cigarettes, 10% currently smoked and 31% of ever-smokers reported less than 10 pack-years. Twenty-seven percent had airflow limitation and 10% had emphysema on CT. The mean TPVV was 130.7±34.9 cm^3^.

Ever-smokers were more likely to be white or African-American males, to have hypertension, airflow limitation, and greater LV mass index and LV mass/end-diastolic volume ratio compared to never-smokers (Table 1). Percent TPVV was similar in the two groups; after adjustment for CT manufacturer and milliamperes, percent TPVV tended to be lower in ever-smokers compared to never-smokers (2.70 vs. 2.72; P = 0.11).

**Table 1:** Characteristics of MESA lung participants with measurement of LV on cardiac MRI and total pulmonary vascular volume.

	Ever-smokers (N = 1226)	Never-smokers (N = 1077)
**Age, years**	69.3±8.9	68.4±9.2
**Male, no. (%)**	719 (58.6)	398 (37.0)
**Race/ethnicity, no. (%)**		
White	551 (44.9)	361 (33.5)
African-American	357 (29.1)	254 (23.6)
Hispanic	225 (18.4)	224 (20.8)
Chinese-American	93 (7.6)	238 (22.1)
**Body mass index, kg/m**^**2**^	28.3±5.0	27.5±5.2
**Cigarette smoking status, no. (%)**		
Current smoker	220 (17.9)	-
Former smoker	1006 (82.1)	-
**Pack-years**^**a**^	26.6±26.2	-
10 or fewer pack-years, no (%)	337 (31.1)	-
Greater than 10 pack-years, no (%)	747 (68.9)	-
**Hypertension, no. (%)**	729 (59.5)	612 (56.8)
**Systolic blood pressure, mmHg**	122.4±19.8	123.3±19.6
**Total cholesterol, mmol/L**	4.65±1.0	4.87±0.9
**HDL cholesterol, mmol/L**	1.42±0.4	1.47±0.4
**Triglycerides, mmol/L**	1.23±0.7	1.24±0.7
**Diabetes, no. (%)**	222 (18.2)	186 (17.4)
**Fasting glucose, mmol/L**	5.6±1.4	5.5±1.3
**Serum creatinine, μmol/L**	82.2±22.1	76.9±26.5
**Diuretic use, no. (%)**	335 (27.3)	237 (22.0)
**Airflow limitation, no. (%)**^**b**^	352 (31.3)	208 (21.3)
**Percent emphysema, median (IQR)**	1.83 (0.69, 3.84)	1.11 (0.48, 2.54)
**Percent predicted total lung volume on CT**	1.05±0.18	1.02±0.16
**Report of dyspnea on exertion, no. (%)**	306 (25.0)	237 (22.2)
**Report of wheezing, no. (%)**	172 (14.0)	81 (7.5)
**Report of chronic cough, no. (%)**	135 (11.0)	85 (7.9)
**TPVV, cm**^**3**^ **vessel**	137.7±34.9	122.6±33.1
**Percent TPVV, % of lung volume**	2.70±0.27	2.71±0.26
**Heart rate, bpm**^**c**^	65.7±11.0	66.9±11.2
**LV end-diastolic volume index, mL/m**^**2**^	64.8±14.9	64.0±12.7
**Stroke volume index, mL/m**^**2**^	39.4±8.6	39.8±8.1
**Cardiac index, L/min/m**^**2**^^**c**^	2.6±0.6	2.6±0.6
**LV mass index, g/m**^**2**^	68.5±14.5	64.1±12.7
**LV mass/end-diastolic volume ratio, g/mL**	1.09±0.25	1.02±0.21
**LV ejection fraction, %**	61.4±7.4	62.5±7.1
**Left atrial volume index, mL/m**^**2**^^**d**^	36.4±11.1	36.3±11.0
**Peak early diastolic strain rate, %/msec**^**e**^	0.11±0.06	0.13±0.06
**Strain relaxation index, msec/%**^**f**^	2.34±1.74	2.09±1.53
**25-min post-contrast T**_**1**_ **time, msec**^**g**^	520±40	518±43
**Extracellular volume fraction, %**^**h**^	26.8±3.1	26.9±3.0

Data are presented as no. (%) or mean±SD, except as noted. Abbreviations: HDL, high density lipoprotein; IQR, interquartile range; TPVV, total pulmonary vascular volume.

^a^ Among 1084 ever-smokers reporting pack-years.

^b^ Airflow limitation defined as pre-bronchodilator FEV_1_/FVC <0.7.

^c^ Among 1152 ever-smokers, 1018 never-smokers.

^d^ Among 1056 ever-smokers, 943 never-smokers.

^e^Among 997 ever-smokers and 1055 never-smokers.

^f^Among 926 ever-smokers and 1000 never-smokers.

^g^ Among 548 ever-smokers and 420 never-smokers.

^h^ Among 238 ever-smokers and 169 never-smokers.

Across quintiles, lower values of percent TPVV were associated with greater age, BMI and percent emphysema, white and Chinese-American race/ethnicity, current smoking, hypertension, diuretic use, airflow limitation (S2 Table).

### Total pulmonary vascular volume and LV filling

Among all participants, a lower TPVV was associated with decrements in LV end-diastolic volume (fully-adjusted effect estimate -5.12mL/SD TPVV [95% CI, -6.80 to -3.43, P-value<0.001]). The association between TPVV and LV end-diastolic volume was modified by smoking history (P-interaction = 0.03); results were therefore stratified by smoking.

Among ever-smokers, a lower TPVV was associated with decrements in LV end-diastolic volume, stroke volume and cardiac output (Table 2). A lower TPVV was associated with a lower LV mass and greater LV mass/end-diastolic volume ratio but not with LV ejection fraction.

**Table 2:** Mean differences in LV parameters associated with a 1 SD lower total pulmonary vascular volume, stratified by smoking status.

	Ever-smokers (N = 1226) Estimate (95% CI)	P-value	Never-smokers (N = 1077) Estimate (95% CI)	P-value
**LV end-diastolic volume, mL**				
Model 1	-4.44 (-6.64, -2.24)	<0.001	-1.18 (-3.42, 1.06)	0.30
Model 2	-6.88 (-9.18, -4.58)	<0.001	-2.71 (-5.18, -0.24)	0.03
**Stroke volume, mL**				
Model 1	-3.08 (-4.41, -1.75)	<0.001	-0.45 (-1.93, 1.02)	0.55
Model 2	-4.54 (-5.93, -3.15)	<0.001	-1.46 (-3.08, 0.16)	0.08
**Cardiac output, L/min**^a^				
Model 1	-0.15 (-0.24, -0.06)	0.002	-0.06 (-0.17, 0.05)	0.30
Model 2	-0.24 (-0.34, -0.14)	<0.001	-0.10 (-0.22, 0.02)	0.09
**LV mass, g**				
Model 1	-1.37 (-3.27, 0.52)	0.16	-0.73 (-2.69, 1.24)	0.47
Model 2	-4.65 (-6.59, -2.71)	<0.001	-2.26 (-4.37, -0.14)	0.04
**LV mass/end-diastolic volume ratio, g/mL**				
Model 1	0.029 (0.010, 0.048)	0.003	0.006 (-0.013, 0.025)	0.54
Model 2	0.025 (0.005, 0.044)	0.01	0.005 (-0.017, 0.026)	0.67
**LV ejection fraction, %**				
Model 1	-0.41 (-0.99, 0.17)	0.16	0.51 (-0.17, 1.19)	0.14
Model 2	-0.51 (-1.14, 0.12)	0.12	0.32 (-0.44, 1.08)	0.41

Model 1: Adjusted for age, sex, race/ethnicity, height, weight, education, CT scanner manufacturer and milliamperes. Model 2: Additionally adjusted for total cholesterol, high density lipoprotein cholesterol, triglycerides, hypertension, systolic blood pressure, diabetes, fasting glucose, creatinine, diuretic use, percent predicted FEV_1_ and percent emphysema, as well as current smoking status and pack-years for ever-smokers

^a^ Cardiac output available for 1152 ever-smokers and 1018 never-smokers.

**Fig 2:**
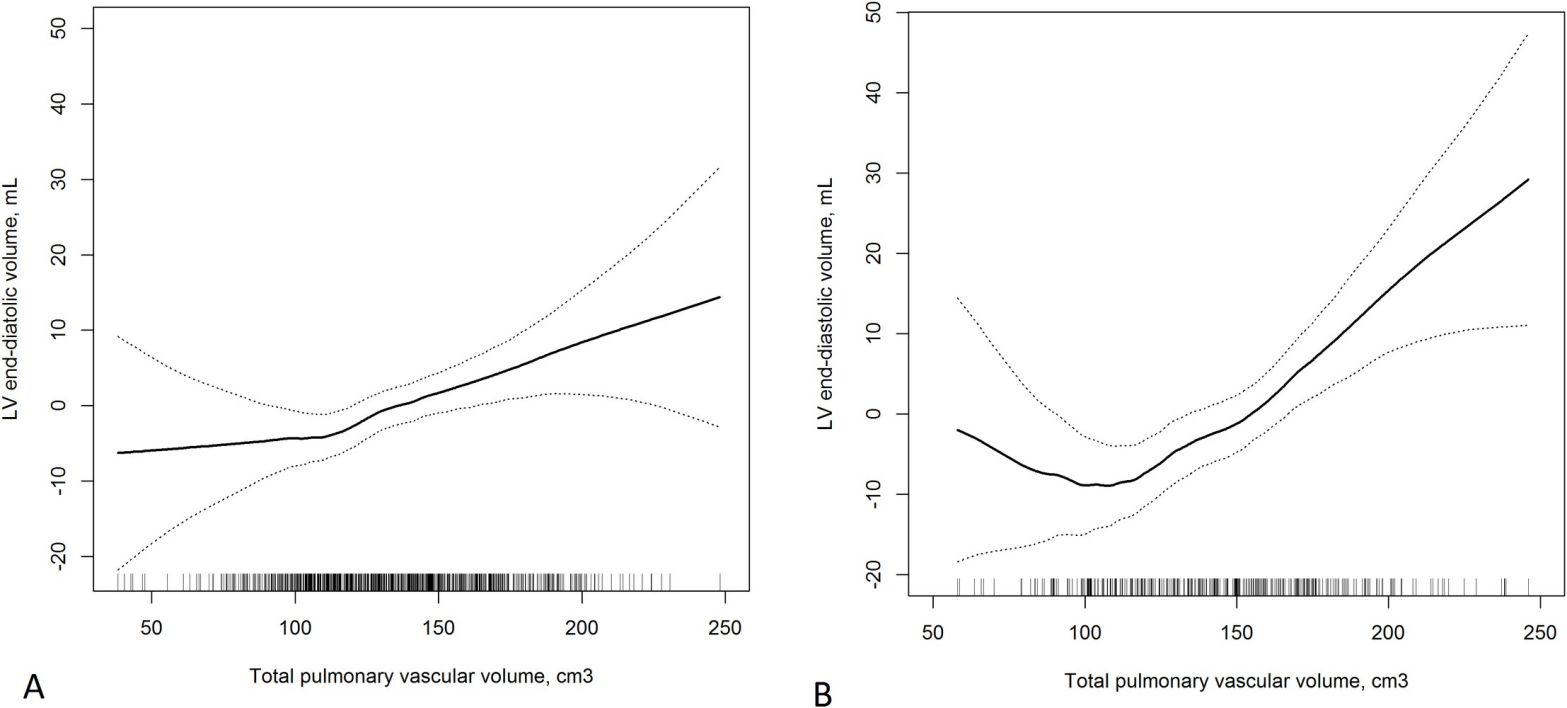
**Generalized additive multivariate model of relationship between total pulmonary vascular volume and LV end-diastolic volume among ever-smokers (A) without airflow limitation and (B) with airflow limitation.** Results are adjusted for age, sex, race/ethnicity, height, weight, education, CT scanner manufacturer and milliamperes, current smoking, pack-years, total cholesterol, high density lipoprotein cholesterol, triglycerides, hypertension, systolic blood pressure, diabetes, fasting glucose, creatinine, diuretic use and percent emphysema. (Missing data for Fig 2 follows a single imputation approach.) Panel A (Without airflow limitation): N = 771, P-linearity<0.001, P-nonlinearity = 0.59. Panel B (With airflow limitation): N = 352, P-linearity = 0.02, P-nonlinearity<0.001. The increase in LV EDV seen at lower values of TPVV flattens after adjustment for LV ejection fraction.

The association was similar among those with and without airflow limitation (Fig 2) and emphysema (S2 Fig), and the association was approximately linear across TPVV values. Additionally, the association among those smoking 0–10 pack-years was of similar magnitude as for those smoking more than 10 pack-years (S3 Fig). Results for percent TPVV were similar (S3 Table).

Among never-smokers, there were smaller magnitude associations between TPVV and LV end-diastolic volume and LV mass; findings were null for other LV parameters (Table 2).

Findings for LV end-diastolic volume among ever-smokers were consistent across strata of sex, race/ethnicity, age, smoking, hypertension, diabetes and LV ejection fraction, with stronger associations among current smokers and those without diabetes (S4 Fig). The association was also significant among ever-smokers without asthma, without a restrictive ventilatory defect and without a measured or self-reported lung disease (S4 Fig). Additional adjustment for FEV_1_/FVC ratio, high attenuation areas on CT, LV ejection fraction, pulse oximetry, total body water, physical activity, coronary artery calcium, and medication use had little impact on the results (S4 Fig), while there was a greater magnitude association after adjustment for percent predicted total lung volume on CT.

### Total pulmonary vascular volume, left atrial volume, diastolic strain and myocardial fibrosis

A lower TPVV was associated with lower left atrial volume overall (-3.28 mL/SD TPVV, P-value<0.001) and among ever-smokers (S4 Table). There was no evidence for a relationship of TPVV to peak early diastolic strain rate or strain relaxation index (S4 Table). Among ever-smokers a lower TPVV was associated with a shorter post-contrast T_1_ time and a higher extracellular volume (ECV) fraction, the latter significant only in the fully-adjusted model, while results for never-smokers were non-significant (S4 Table).

### Pulmonary microvascular blood volume and LV filling, left atrial volume and pulmonary vein area

In a second sample of 142 ever-smokers, 56% of whom had COPD (full characteristics in S5 Table), lower pulmonary microvascular blood volume on MRI was associated with lower LV end-diastolic volume, stroke volume and pulmonary vein area, and greater LV mass/end-diastolic volume ratio (S6 Table). Results remained significant after adjustment for RV parameters.

### Total pulmonary vascular volume and respiratory symptoms

Twenty-three percent of participants reported dyspnea, 11% reported wheezing and 9% chronic cough. In the overall sample, a lower TPVV was associated with dyspnea in unadjusted and fully-adjusted analyses (OR 1.24/SD TPVV; P-value = 0.02). The association between TPVV and dyspnea was also modified by smoking history (P-interaction = 0.03).

Among ever-smokers, a lower TPVV was associated with dyspnea and wheezing independent of lung function, percent emphysema and LV ejection fraction (Table 3). Associations of TPVV with dyspnea and wheezing persisted among ever-smokers without airflow limitation or emphysema (dyspnea: OR 1.55/SD TPVV, P-value = 0.01; wheezing: OR 1.75/SD TPVV, P-value = 0.01). There were no associations between TPVV and respiratory symptoms among never-smokers in adjusted models.

**Table 3:** Association of respiratory symptoms with 1 standard deviation lower total pulmonary vascular volume stratified by smoking history.

	Ever-smokers (N = 1226) Odds Ratio (95% CI)	P-value	Never-smokers (N = 1074) Odds Ratio (95% CI)	P-value
**Dyspnea**^a^				
Unadjusted	1.61 (1.40, 1.85)	<0.001	1.37 (1.17, 1.62)	<0.001
Model 1	1.58 (1.28, 1.94)	<0.001	1.03 (0.80, 1.34)	0.80
Model 2	1.46 (1.15, 1.85)	0.002	0.92 (0.69, 1.23)	0.58
**Wheezing**				
Unadjusted	1.35 (1.14, 1.59)	<0.001	1.34 (1.04, 1.73)	0.03
Model 1	1.59 (1.24, 2.04)	<0.001	1.14 (0.77, 1.69)	0.51
Model 2	1.41 (1.06, 1.87)	0.02	0.89 (0.56, 1.40)	0.61
**Chronic cough**				
Unadjusted	1.09 (0.91, 1.30)	0.36	1.15 (0.90, 1.46)	0.26
Model 1	1.00 (0.76, 1.32)	0.997	1.06 (0.71, 1.56)	0.79
Model 2	0.94 (0.69, 1.28)	0.69	0.85 (0.55, 1.33)	0.49

Model 1: adjusted for age, sex, race/ethnicity, height, weight, CT manufacturer and milliamperes, and current smoking and pack-years for ever-smokers. Model 2: additionally adjusted for percent predicted FEV_1_, percent emphysema and LV ejection fraction

^a^ Dyspnea was reported for 1222 ever-smokers and 1065 never-smokers.

## Discussion

In this large, population-based study, a lower pulmonary vascular volume was associated with decrements in left atrial volumes, LV end-diastolic volume, stroke volume and cardiac output, and with dyspnea among persons who had smoked more than 100 lifetime cigarettes, including those without demonstrable smoking-related lung disease. These findings suggest that subtle changes in the pulmonary vasculature–which are generally unobserved in clinical practice–may impair LV filling and contribute to respiratory symptoms in ever-smokers in the general population in the absence of impaired LV relaxation.

This is the first study of which we are aware to assess the pulmonary vasculature, cardiac function and dyspnea in a population-based sample. Small studies have correlated pulmonary blood volume with cardiac output and LV stroke volume in valvular heart disease [40, 41], and with LV end-diastolic volume index in severe emphysema [42]. Dyspnea on exertion has been attributed to the inability to augment pulmonary blood flow during exercise in pulmonary arterial hypertension [43, 44] and tricuspid regurgitation [45]. In addition, a lower volume of small pulmonary vessels on CT was correlated with reduced six minute walk distance in smokers, most of whom had COPD [10].

A potential mechanism for these findings is diffuse, smoking-related pulmonary microvascular damage leading to reduced pulmonary blood flow, causing reduced inflow to the LV. Smaller pulmonary capillary volumes are seen in smoke-exposed animals [46] and pulmonary arterial remodeling occurs in smokers [47]. As the pulmonary circulation accommodates high blood flow (~5–6 L/minute) over a low pressure gradient (~10 mmHg), minor but diffuse damage to the pulmonary vasculature may reduce blood flow to the left heart. Another possibility is that other smoking-related pulmonary changes, such as small airways disease-associated hyperinflation compressing the pulmonary vasculature [29, 48], or regional hypoxic vasoconstriction [49], may have contributed to the findings.

An alternative hypothesis is that the association of TPVV with reduced LV filling was due to increased LV stiffness with increased LV end-diastolic pressure (i.e., impaired LV relaxation or “diastolic dysfunction”). A lower TPVV was associated with concentric LV remodeling (i.e., greater LV mass/end-diastolic volume ratio), which suggests impaired LV relaxation [50], however, this finding was due exclusively to the decrement in LV end-diastolic volume. Additionally, a lower TPVV was associated with a lower, not greater, left atrial volume, and there was no association with impaired LV relaxation on MRI. Furthermore, reduced LV filling due to LV stiffness would be expected to increase pulmonary blood volume, not decrease it. Finally, a lower pulmonary microvascular blood volume was associated with smaller pulmonary veins, further suggesting low-to-normal LV end-diastolic pressures, and no signs of impaired LV relaxation on MRI. Hence, the finding for LV concentric remodeling is likely a false positive sign for impaired LV relaxation in this setting. In conjunction with dyspnea in some patients, this finding could be mistakenly attributed to HFpEF.

Nonetheless, the findings of a shorter 25-minute post-contrast T_1_ time and greater extracellular volume fraction suggest that those with a lower TPVV have greater myocardial fibrosis. While LV fibrosis occurs in HFpEF and correlates with LV stiffness [51], one study found a parallel reduction in myocardial microvascular density [52] suggesting that LV fibrosis may be secondary to systemic microvascular damage seen in smokers [53, 54]. Nevertheless, the cumulative results from our general population sample suggest that a lower TPVV is associated with a phenotype of impaired cardiac filling without an increase in LV end-diastolic pressure (and thus distinct from HFpEF); the potential contribution of LV fibrosis to this phenotype deserves further study.

Notably, the association between TPVV and LV filling was independent of measured lung function, percent emphysema and high attenuation areas on CT and was present among participants without functional or structural evidence of smoking-related lung disease. These findings suggest that smoking-related changes in the pulmonary vasculature might contribute to impaired cardiac filling and dyspnea independent of, and in the absence of, lung disease. This may have potential implications for disease prevention, as some early pulmonary vascular changes appear reversible [55].

A lower TPVV was associated with decrements in LV end-diastolic volume even among participants who had smoked 0–10 pack-years. This suggests a risk to the pulmonary vasculature associated with a smoking history that is usually considered insignificant for smoking-related lung disease, but is consistent with increased cardiovascular risk observed in light and intermittent smokers [56].

Strengths of this study include the use of novel quantitative pulmonary vascular measures in a large, multiethnic, general population sample, MRI measures of cardiac structure and function, and precise measures of potential confounders. However, several limitations should be discussed.

First, the TPVV was measured on non-contrast CT and did not assess the microvasculature, pulmonary arterial pressure or flow, and was not validated against invasive measures and is not yet measured in clinical practice. While invasive measures were not feasible in a sample this large, findings were confirmed in a second sample with direct measures of pulmonary microvascular blood volume obtained on contrast-enhanced MRI, measures that were consistent with global lung perfusion and correlated with diffusing capacity [57]. While diffusing capacity was not measured in the larger cohort, it has limited utility for this study as a measure of the pulmonary vasculature as it would be expected to be low with both pulmonary vascular damage and subclinical diastolic dysfunction. Additionally, mean values of TPVV are approximately 25–30% of the pulmonary blood volume measured invasively; this difference is not unexpected, given the included and detectable vessels. While measurement of pulmonary blood volume is not feasible in current practice, the findings in this paper suggest further studies focusing on the pulmonary vasculature as a therapeutic target in HFpEF may be warranted.

Second, TPVV was higher in ever-smokers compared to never-smokers instead of lower as would be expected with the hypothesized microvascular damage incurred by smoking. However, this was largely due to differences in body size, as percent TPVV, normalized to lung volume, tended to be lower in ever-smokers compared to never-smokers. While the microvasculature itself is not included in the TPVV, and we are not able to differentiate arterial from venous volumes, the described associations with a lower TPVV likely reflect a simultaneous reduction in arterial, venous and microvascular volumes.

Third, echocardiography, commonly used to assess diastolic function of the LV, and diastolic stress tests were not performed. However, MRI measures of impaired LV relaxation directly measure strain and predict clinical events [25]. Additionally, results for left atrial volume and pulmonary vein area suggest reduced rather than elevated LV end-diastolic pressure, consistent with the lower estimated left atrial pressure described in COPD [58]. Systemic arterial stiffness, which has been associated with cigarette smoking [59], greater emphysema in COPD [60] and with HFpEF [61, 62] was also not examined, although given the direction of blood flow it would be unlikely for systemic arterial stiffness to alter pulmonary vascular volumes.

Fourth, we were unable to assess the etiology of reduced TPVV or distinguish whether reductions were related to vessel loss, hyperinflation-related compression, hypoxic pulmonary vasoconstriction or RV dysfunction. RV measures were unavailable for the full cohort at this exam, however, RV volumes have been found to be reduced in COPD [27], and were inversely associated with percent emphysema [63] and dyspnea in MESA [64]. Importantly, adjustment for RV measures in the second cohort did not impact the results, suggesting that a smaller RV did not account for the findings. Reduced pulmonary artery distensibility has also been found with increasing severity of COPD [65], a finding that could parallel the reduction in TPVV. Of note, we have also found hyperinflation to be associated with increased LV mass and mass/end-diastolic volume ratio among smokers, many with COPD [66]. However, hyperinflation and hypoxemia are unlikely to fully explain the findings as results were strengthened after adjustment for percent predicted total lung volume on CT, and unchanged after adjustment for pulse oximetry and with restriction to those without evident lung disease.

Fifth, in this cross-sectional analysis, directionality is uncertain and selection bias is possible. It is unlikely that LV causes of impaired filling contributed to reductions in pulmonary vascular volume, and results were unchanged after adjustment for LV ejection fraction. The study was population-based and characteristics of the current sample were only modestly different from the overall sample, making selection bias an unlikely explanation for the results.

Finally, confounding is a concern in any observational study and residual confounding by smoking, hypertension and body size is possible. However, major confounders were measured precisely: current smoking was verified by urinary cotinine, blood pressure and medications were measured directly. In addition, results were consistent using TPVV indexed to lung volume.

In conclusion, in persons smoking more than 100 lifetime cigarettes a lower TPVV was associated with reduced LV filling and a greater LV mass/end-diastolic volume ratio, and also with increased dyspnea, including among persons without smoking-related lung disease. This suggests that subclinical pulmonary vascular damage may negatively affect cardiac filling and function, and contribute to symptoms in the general population.

## Supporting information

S1 Fig Study population. (TIF)

S2 Fig **Generalized additive multivariate model of relationship between total pulmonary vascular volume and LV end-diastolic volume among ever-smokers (A) without emphysema and (B) with emphysema.** Results are adjusted for age, sex, race/ethnicity, height, weight, education, CT scanner manufacturer, milliamperes, current smoking, pack-years, total cholesterol, high-density lipoprotein cholesterol, triglycerides, hypertension, systolic blood pressure, diabetes, fasting glucose, creatinine, diuretic use, percent predicted FEV_1_. (Missing data for S2 Fig follows a single imputation approach.) Panel A (Without emphysema): N = 1078, P-linearity<0.001, P-nonlinearity = 0.14. Panel B (With emphysema): N = 148, P-linearity = 0.03, P-nonlinearity = 0.41.
(TIF)

S3 Fig Multivariate mean differences in LV end-diastolic volume per standard deviation lower total pulmonary vascular volume stratified by cumulative pack-years of smoking. Results are adjusted for age, sex, race/ethnicity, height, weight, education, CT scanner manufacturer, milliamperes, total cholesterol, high-density lipoprotein cholesterol, triglycerides, hypertension, systolic blood pressure, diabetes, fasting glucose, creatinine, diuretic use, percent predicted FEV_1_ and percent emphysema, as well as current smoking status for ever-smokers.
(TIF)

S4 Fig Sensitivity analysis for mean difference in LV end-diastolic volume (mL) per 1 standard deviation lower total pulmonary vascular volume for ever-smokers. Abbreviations: LV EF = left ventricular ejection fraction, FEV_1_ = forced expiratory capacity in 1 second, FVC = forced vital capacity, ACE = angiotensin converting enzyme, ARB = angiotensin II receptor blocker. *Lung disease includes airflow limitation (FEV_1_/FVC<0.7), restrictive ventilatory defect (FVC< lower limit of normal and FEV_1_/FVC>0.7), emphysema above the upper limit of normal, and self-report of COPD, emphysema, asthma or pulmonary fibrosis. Results are adjusted for age, sex, race/ethnicity, height, weight, education, CT scanner manufacturer, milliamperes, current smoking, pack-years, total cholesterol, high-density lipoprotein cholesterol, triglycerides, hypertension, systolic blood pressure, diabetes, fasting glucose, creatinine, diuretic use, percent predicted FEV_1_ and percent emphysema. P-values for interactions: sex 0.19; race/ethnicity 0.33; age 0.34; current vs. former smoking 0.01; hypertension 0.72; diabetes 0.001; LV ejection fraction 0.34; asthma 0.63, restrictive ventilator defect 0.43; any lung disease 0.33.
(TIF)

S1 Table Selected characteristics of MESA Exam 5 participants included and not included in this analysis. Data are presented as no. (%) or mean±SD, except as noted. Abbreviations: IQR, interquartile range; FEV_1_, forced expiratory volume in 1 second; FVC, forced vital capacity; TPVV, total pulmonary vascular volume; LV, left ventricular. *Among ever-smokers reporting pack-years, 1085 included and 826 not included in this analysis. ^†^Among those with spirometry, 2101 included and 1021 not included in the analysis. Airflow limitation defined as pre-bronchodilator FEV_1_/FVC < 0.7. ^‡^Among 828 participants who underwent full-lung CT but not cardiac MRI. ^§^Among 797 MESA Exam 5 participants who underwent cardiac MRI but not full-lung CT. ^ll^Among 2170 included in this analysis. **Among 1999 included and 605 not included in this analysis. ^††^Among 2052 included and 697 not included in this analysis. ^‡‡^Among 1926 included and 630 not included in this analysis.
(PDF)

S2 Table Selected characteristics of included participants by quintile of *percent* total pulmonary vascular volume. Data are presented as % or mean±SD, except as noted. Abbreviations: IQR, interquartile range; FEV_1_, forced expiratory volume in 1 second; FVC, forced vital capacity; TPVV, total pulmonary vascular volume. *For ever-smokers reporting pack-years, N = 239, 211, 217, 202 and 215 across quintiles. ^†^Airflow limitation defined as pre-bronchodilator FEV_1_/FVC < 0.7.
(PDF)

S3 Table Mean differences in LV parameters associated with a 1 standard deviation lower *percent* total pulmonary vascular volume, stratified by smoking status. Model 1: Adjusted for age, sex, race/ethnicity, height, weight, education, CT scanner manufacturer and milliamperes. Model 2: Additionally adjusted for total cholesterol, high-density lipoprotein cholesterol, triglycerides, hypertension, systolic blood pressure, diabetes, fasting glucose, creatinine, diuretic use, percent predicted FEV_1_ and percent emphysema, as well as current smoking status and pack-years for ever-smokers. *Cardiac output available for 1152 ever-smokers and 1018 never-smokers.
(PDF)

S4 Table Mean differences in left atrial volume, peak early diastolic strain rate, strain relaxation index, T_1_ time and extracellular volume fraction associated with a 1 standard deviation lower total pulmonary vascular volume on computed tomography, stratified by smoking status. Model 1: Adjusted for age, sex, race/ethnicity, height, weight, education, CT scanner manufacturer and milliamperes. Model 2: Additionally adjusted for total cholesterol, high density lipoprotein cholesterol, triglycerides, hypertension, systolic blood pressure, diabetes, fasting glucose, creatinine, diuretic use, percent predicted FEV_1_ and percent emphysema, as well as current smoking status and pack-years for ever-smokers. *T_1_ time and extracellular volume fraction models also adjusted for heart rate and left ventricular end-diastolic mass.
(PDF)

S5 Table Characteristics of participants with LV parameters and pulmonary microvascular blood volume measured on MRI. Data are presented as % or mean±SD, except as noted. Abbreviations: HDL, high-density lipoprotein; COPD, chronic obstructive pulmonary disease; GOLD, Global initiative for chronic obstructive lung disease; IQR, interquartile range. *Calculated as pulmonary microvascular blood volume (cm^3^ blood/100 cm^3^ lung) x total lung volume on CT scans obtained at functional residual capacity, in 91 subjects.
(PDF)

S6 Table Mean differences in LV parameters, left atrial volume and pulmonary vein area associated with a 1 SD lower pulmonary microvascular blood volume on MRI in an independent sample of ever-smokers (N = 142). Abbreviations: LV, left ventricular; EDV, end-diastolic volume. Model 1: Adjusted for age, sex, race/ethnicity, height, weight, education and cohort. Model 2: Additionally adjusted for smoking status, pack-years, total cholesterol, high density lipoprotein cholesterol, triglycerides, hypertension, systolic blood pressure, diabetes, fasting glucose, creatinine, diuretic use, percent predicted FEV_1_ and percent emphysema. Model 3: Additionally adjusted for respective right ventricular parameter. *N = 141, 1 participant with uninterpretable right ventricular measures on MRI. ^†^N = 139, 3 participants without left atrial volume or pulmonary vein area measurement. ^‡^Adjusted for right ventricular end-diastolic volume. N = 138, 1 participant with uninterpretable right ventricular measures on MRI.
(PDF)
