# Supplementary figures and images for: Pulmonary vascular volume, impaired left ventricular filling and dyspnea: The MESA Lung Study
Source: PLoS One. 2017 Apr 20;12(4):e0176180. doi: 10.1371/journal.pone.0176180 (PMC5398710; doi:10.1371/journal.pone.0176180)

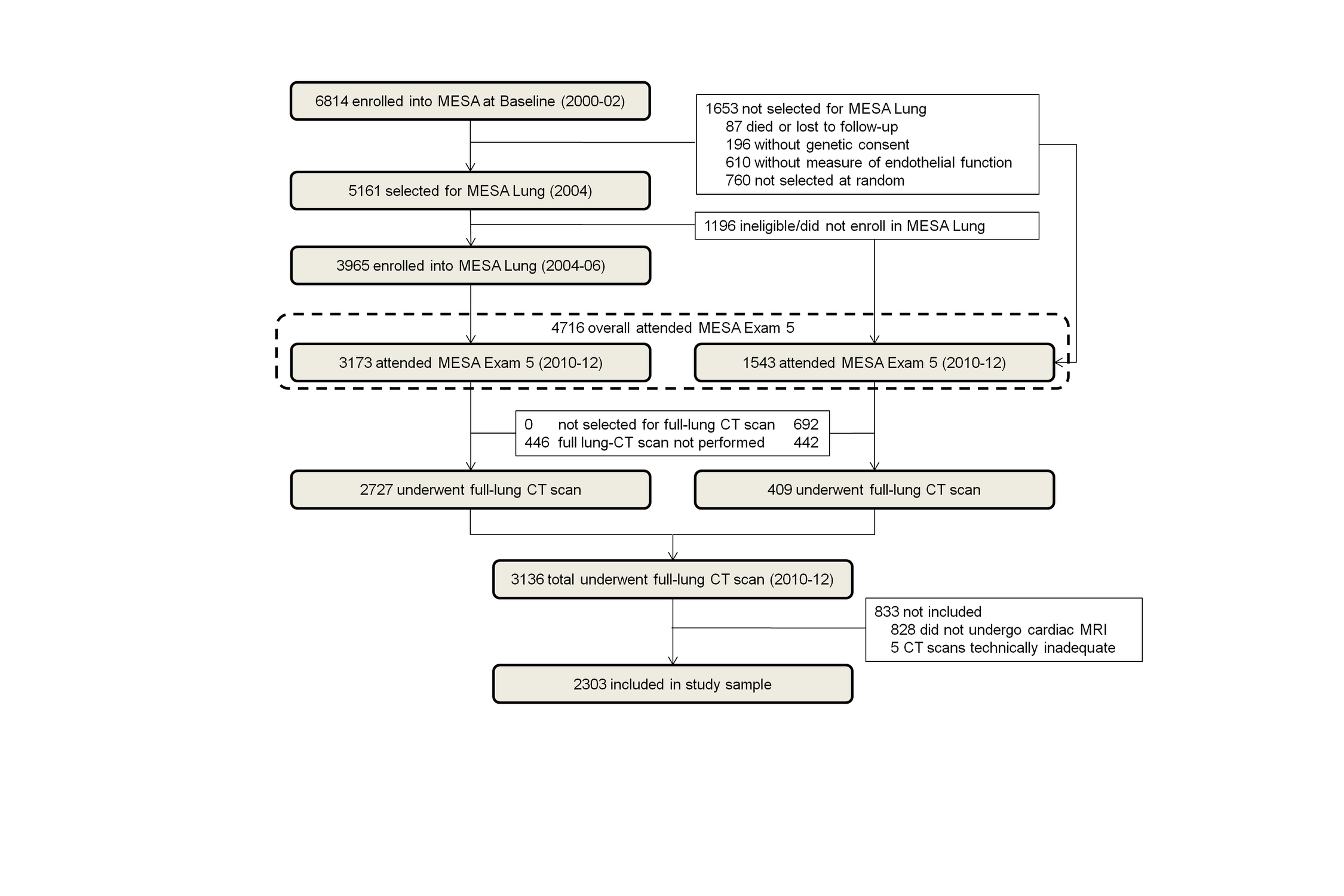

Supplement: S1 Fig — (TIF) [file pone.0176180.s001.tif]

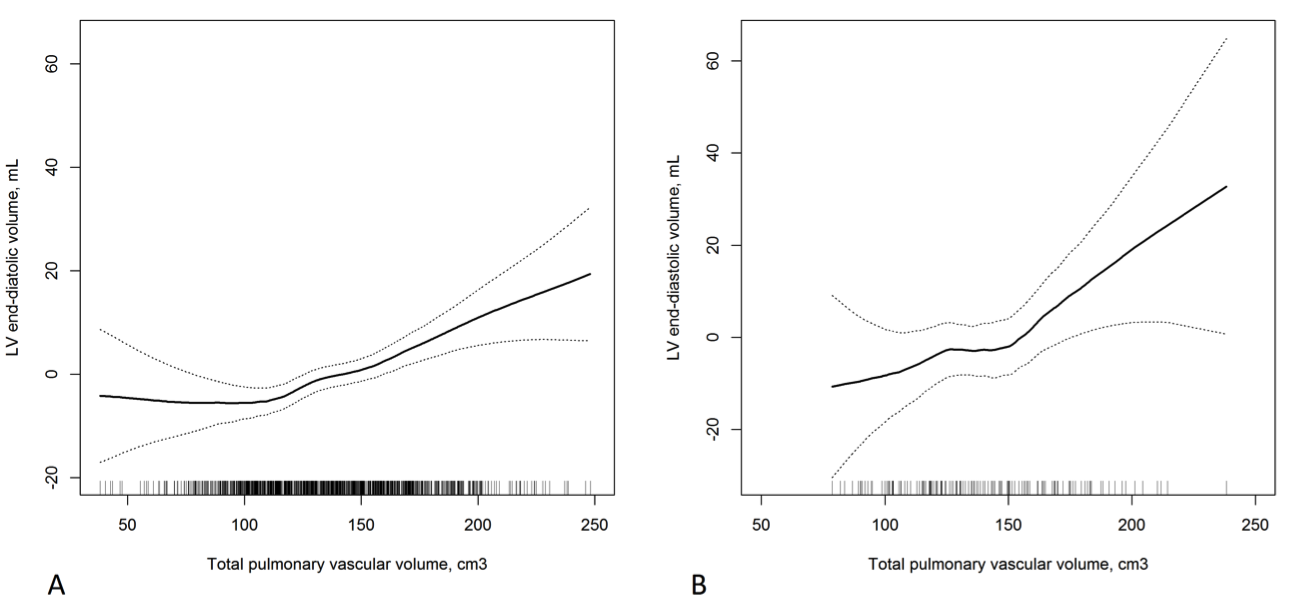

Supplement: S2 Fig — Generalized additive multivariate model of relationship between total pulmonary vascular volume and LV end-diastolic volume among ever-smokers (A) without emphysema and (B) with emphysema. Results are adjusted for age, sex, race/ethnicity, height, weight, education, CT scanner manufacturer, milliamperes, current smoking, pack-years, total cholesterol, high-density lipoprotein cholesterol, triglycerides, hypertension, systolic blood pressure, diabetes, fasting glucose, creatinine, diuretic use, percent predicted FEV1. (Missing data for S2 Fig follows a single imputation approach.) Panel A (Without emphysema): N = 1078, P-linearity<0.001, P-nonlinearity = 0.14. Panel B (With emphysema): N = 148, P-linearity = 0.03, P-nonlinearity = 0.41. (TIF) [file pone.0176180.s002.tif]

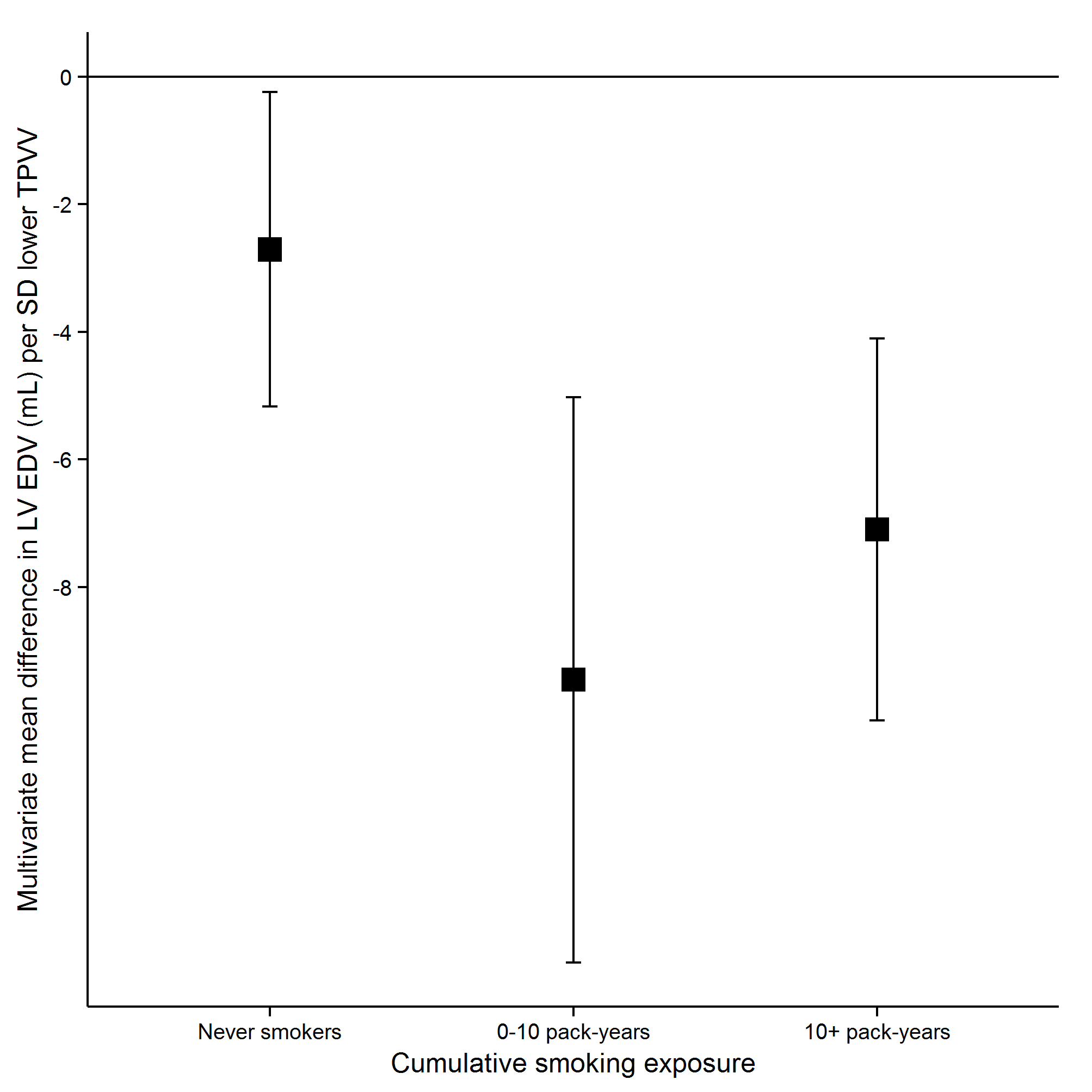

Supplement: S3 Fig — Results are adjusted for age, sex, race/ethnicity, height, weight, education, CT scanner manufacturer, milliamperes, total cholesterol, high-density lipoprotein cholesterol, triglycerides, hypertension, systolic blood pressure, diabetes, fasting glucose, creatinine, diuretic use, percent predicted FEV1 and percent emphysema, as well as current smoking status for ever-smokers. (TIF) [file pone.0176180.s003.tif]

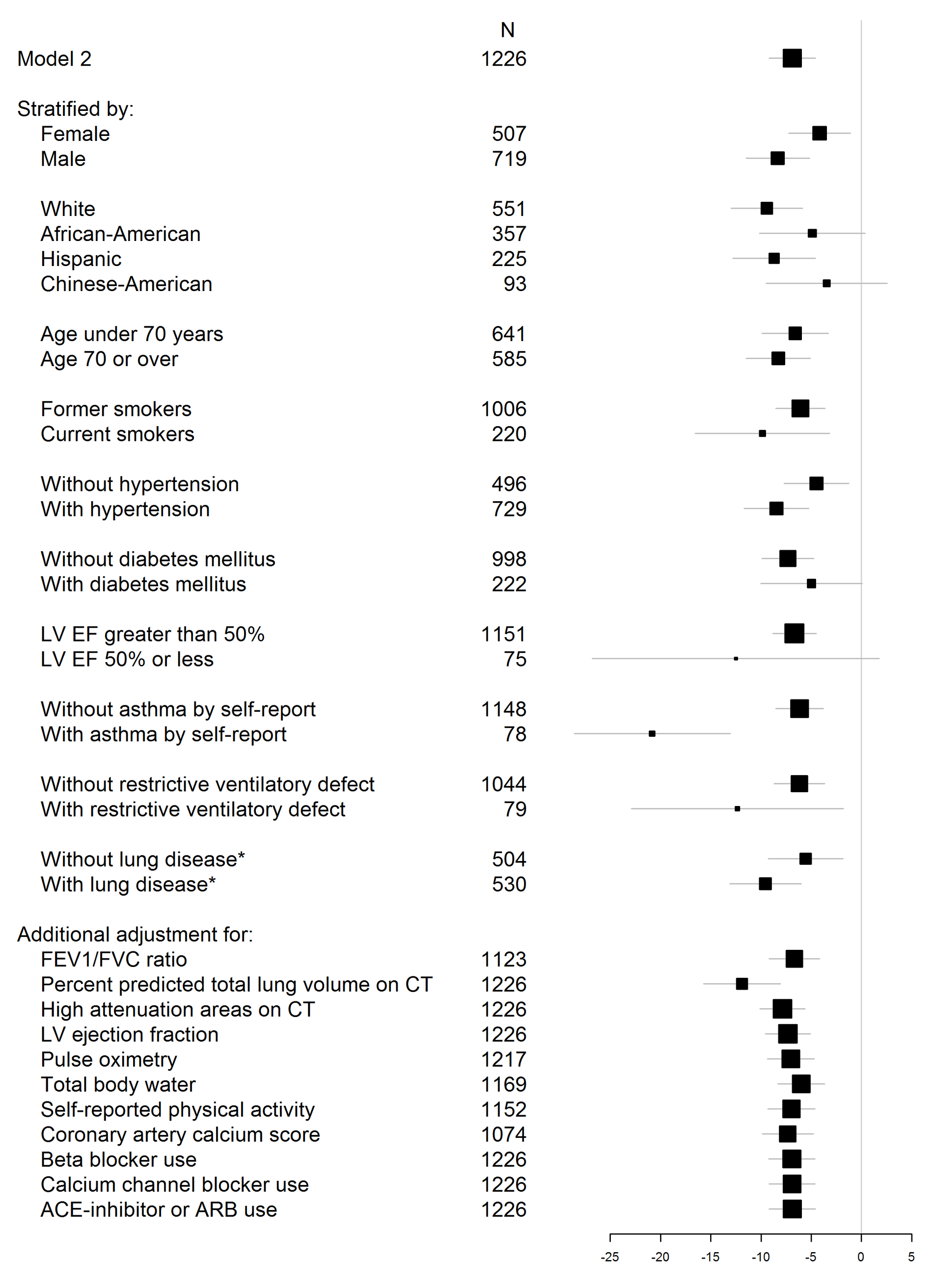

Supplement: S4 Fig — Abbreviations: LV EF = left ventricular ejection fraction, FEV1 = forced expiratory capacity in 1 second, FVC = forced vital capacity, ACE = angiotensin converting enzyme, ARB = angiotensin II receptor blocker. *Lung disease includes airflow limitation (FEV1/FVC<0.7), restrictive ventilatory defect (FVC< lower limit of normal and FEV1/FVC>0.7), emphysema above the upper limit of normal, and self-report of COPD, emphysema, asthma or pulmonary fibrosis. Results are adjusted for age, sex, race/ethnicity, height, weight, education, CT scanner manufacturer, milliamperes, current smoking, pack-years, total cholesterol, high-density lipoprotein cholesterol, triglycerides, hypertension, systolic blood pressure, diabetes, fasting glucose, creatinine, diuretic use, percent predicted FEV1 and percent emphysema. P-values for interactions: sex 0.19; race/ethnicity 0.33; age 0.34; current vs. former smoking 0.01; hypertension 0.72; diabetes 0.001; LV ejection fraction 0.34; asthma 0.63, restrictive ventilator defect 0.43; any lung disease 0.33. (TIF) [file pone.0176180.s004.tif]
