# Supplementary material for: Pulmonary vascular volume, impaired left ventricular filling and dyspnea: The MESA Lung Study
Source: PLoS One. 2017 Apr 20;12(4):e0176180. doi: 10.1371/journal.pone.0176180 (PMC5398710; doi:10.1371/journal.pone.0176180)
Supplement: S1 Table — Data are presented as no. (%) or mean±SD, except as noted. Abbreviations: IQR, interquartile range; FEV1, forced expiratory volume in 1 second; FVC, forced vital capacity; TPVV, total pulmonary vascular volume; LV, left ventricular. *Among ever-smokers reporting pack-years, 1085 included and 826 not included in this analysis. †Among those with spirometry, 2101 included and 1021 not included in the analysis. Airflow limitation defined as pre-bronchodilator FEV1/FVC < 0.7. ‡Among 828 participants who underwent full-lung CT but not cardiac MRI. §Among 797 MESA Exam 5 participants who underwent cardiac MRI but not full-lung CT. llAmong 2170 included in this analysis. **Among 1999 included and 605 not included in this analysis. ††Among 2052 included and 697 not included in this analysis. ‡‡Among 1926 included and 630 not included in this analysis. (PDF) [file pone.0176180.s005.pdf]

	Included (N=2303)	Not included (N=2413)
Age, years	68.9±9.0	70.9±9.8
Male, no. (%)	1117 (48.5)	1085 (45.0)
Race/ethnicity, no. (%)		
White	912 (39.6)	1014 (42.0)
African-American	611 (26.5)	639 (26.5)
Hispanic	449 (19.5)	550 (22.8)
Chinese-American	331 (14.4)	210 (8.7)
Education, no. (%)		
No high school degree	286 (12.4)	386 (16.0)
High school degree	397 (17.2)	425 (17.6)
Some college	645 (28.0)	726 (30.1)
College degree	453 (19.7)	417 (17.3)
Graduate school	518 (22.5)	454 (18.8)
Body mass index, kg/m^2	28.0±5.1	29.0±6.1
Cigarette smoking status, no. (%)		
Never smoker	1077 (46.8)	1204 (51.3)
Current smoker	220 (9.6)	176 (7.5)
Former smoker	1006 (43.7)	950 (40.0)
Pack-years for ever-smokers*	26.6±26.2	23.4±23.2
Hypertension, no. (%)	1341 (58.3)	1437 (61.1)
Total cholesterol, mmol/L	4.76±1.0	4.71±1.0
Diabetes, no. (%)	408 (17.8)	513 (22.2)
Fasting glucose, mmol/L	5.57±1.4	5.77±1.8
Serum creatinine, μmol/L	80.4±30.9	83.1±39.8
Diuretic use, no. (%)	572 (24.8)	682 (28.3)
FEV_1/FVC ratio[†]	0.74±0.09	0.74±0.09
Airflow limitation, no. (%)[†]	560 (26.7)	286 (28.0)
Percent emphysema, median (IQR)	1.48 (0.57, 3.25)	1.17 (0.49, 2.69)
TPVV, cm^3	130.7±34.9	123.9±34.7[‡]
Percent TPVV, %	2.71±0.27	2.69±0.28[‡]
LV end diastolic volume index, mL/m^2	64.4±13.9	64.6±14.0[§]
Stroke volume index, mL/m^2	39.6±8.4	40.0±8.8[§]
Cardiac index, L/min/m^2	2.6±0.6[ll]	2.6±0.6[§]
LV mass index, g/m^2	66.4±13.9	65.4±13.2[§]
LV mass/end-diastolic volume ratio, g/mL	1.06±0.23	1.04±0.23[§]
LV ejection fraction, %	61.8±7.3	62.2±7.2[§]
Left atrial volume index, mL/m^2**	36.3±10.8	36.8±11.6
Peak early diastolic strain rate, %/msec[††]	0.12±0.06	0.11±0.05
Strain relaxation index, msec/%[‡‡]	2.21±1.64	2.27±1.80
