# Supplementary material for: Pulmonary vascular volume, impaired left ventricular filling and dyspnea: The MESA Lung Study
Source: PLoS One. 2017 Apr 20;12(4):e0176180. doi: 10.1371/journal.pone.0176180 (PMC5398710; doi:10.1371/journal.pone.0176180)
Supplement: S2 Table — Data are presented as % or mean±SD, except as noted. Abbreviations: IQR, interquartile range; FEV1, forced expiratory volume in 1 second; FVC, forced vital capacity; TPVV, total pulmonary vascular volume. *For ever-smokers reporting pack-years, N = 239, 211, 217, 202 and 215 across quintiles. †Airflow limitation defined as pre-bronchodilator FEV1/FVC < 0.7. (PDF) [file pone.0176180.s006.pdf]

	Quintile 1 (N=460)	Quintile 2 (N=461)	Quintile 3 (N=461)	Quintile 4 (N=461)	Quintile 5 (N=460)
Percent TPVV, %	2.33±0.14	2.57±0.04	2.71±0.04	2.84±0.04	3.08±0.13
Age, years	72.2±9.2	68.9±8.9	69.5±9.0	67.5±8.8	66.4±8.3
Male, no. (%)	222 (48.3)	208 (45.1)	231 (50.1)	228 (49.5)	228 (49.6)
Race/ethnicity, no. (%)					
White	199 (43.3)	185 (45.1)	193 (41.9)	175 (38.0)	160 (34.8)
African-American	128 (27.8)	118 (25.6)	119 (25.8)	118 (25.6)	128 (27.8)
Hispanic	59 (12.8)	85 (18.4)	79 (17.1)	107 (23.2)	119 (25.9)
Chinese-American	74 (16.1)	73 (15.8)	70 (15.2)	61 (13.2)	53 (11.5)
Education, no. (%)					
No high school degree	54 (11.7)	58 (12.6)	60 (13.0)	54 (11.7)	60 (13.0)
High school degree	83 (18.0)	84 (18.2)	72 (15.6)	84 (18.2)	74 (16.1)
Some college	130 (28.3)	139 (30.2)	123 (26.7)	134 (29.1)	119 (25.9)
College degree	93 (20.2)	86 (18.7)	85 (18.4)	90 (19.5)	99 (21.5)
Graduate school	98 (21.3)	94 (20.4)	121 (26.2)	97 (21.0)	108 (23.5)
Body mass index, kg/m^2	26.8±4.9	27.7±5.1	28.1±5.0	28.3±5.0	28.8±5.1
Cigarette smoking status, %					
Never-smoker	196 (42.6)	230 (49.9)	210 (45.6)	224 (48.6)	217 (47.2)
Current smoker	54 (11.7)	41 (8.9)	43 (9.3)	46 (10.0)	36 (7.8)
Former smoker	210 (45.7)	190 (41.2)	208 (44.1)	191 (41.4)	207 (45.0)
Pack-years for ever-smokers*	26.8±26.4	25.6±26.1	26.8±24.5	28.0±27.6	26.0±26.7
Hypertension, no (%)	293 (63.7)	270 (58.6)	279 (60.7)	262 (56.8)	237 (51.5)
Systolic blood pressure, mmHg	125.5±19.7	122.9±20.1	122.5±19.8	122.4±19.5	120.9±19.2
Total cholesterol, mmol/L	4.72±0.9	4.82±1.0	4.75±0.9	4.69±0.9	4.80±1.0
HDL cholesterol, mmol/L	1.49±0.4	1.47±0.5	1.42±0.4	1.42±0.4	1.40±0.4
Triglycerides, mmol/L	1.16±0.6	1.30±0.8	1.25±0.7	1.21±0.6	1.24±0.8
Diabetes, no (%)	74 (16.2)	90 (19.6)	85 (18.6)	83 (18.1)	76 (16.6)
Fasting glucose, mmol/L	5.5±1.4	5.6±1.4	5.6±1.4	5.6±1.4	5.6±1.3
Serum creatinine, µmol/L	83.1±25.6	79.6±26.5	81.3±24.8	77.8±22.1	77.8±21.2
Diuretic use, no. (%)	117 (28.5)	116 (27.3)	125 (29.9)	103 (21.7)	111 (16.7)
FEV_1/FVC ratio	0.70±0.11	0.74±0.08	0.73±0.09	0.75±0.07	0.77
Percent predicted FEV_1, %	93.6±22.8	96.7±19.9	96.1±20.5	95.9±17.9	95.4±17.9
Airflow limitation, no. (%)[†]	177 (42.4)	113 (26.8)	122 (29.8)	85 (19.9)	63 (14.8)
Percent emphysema, median (IQR), %	4.93 (1.07, 5.67)	3.29 (0.75, 3.48)	3.00 (0.65, 3.41)	2.15 (0.54, 2.45)	1.63 (0.37, 1.84)
High attenuation areas, cm^3	213.8±65.8	211.5±50.6	218.5±50.8	224.1±46.09	237.2±54.9
TPVV, cm^3	118.7±34.9	125.6±32.2	132.8±35.3	136.9±33.8	139.4±34.0
Pulse oximetry, %	97.0±2.2	97.0±1.6	97.0±1.5	97.1±1.6	97.0±1.8
Total body water, L	37.1±9.4	37.5±9.2	38.6±9.2	39.0±8.9	39.2±8.8
Moderate and vigorous physical activity, met-min/day	721.2±899.8	716.7±714.3	785.7±1060.9	764.7±789.0	817.3±987.6
Coronary artery calcium, Agatston score	309.7±624.1	244.8±459.4	288.2±603.7	221.8±486.4	196.3±439.3
Beta-blocker use, no. (%)	83 (18.0)	89 (19.3)	94 (20.4)	79 (17.1)	65 (14.1)
Calcium channel blocker use, no. (%)	86 (18.7)	75 (16.3)	85 (18.4)	84 (18.2)	74 (16.1)
ACE-inhibitor/ARB use, no. (%)	162 (35.2)	156 (33.84)	163 (35.4)	137 (29.7)	131 (28.5)
