# Supplementary material for: Pulmonary vascular volume, impaired left ventricular filling and dyspnea: The MESA Lung Study
Source: PLoS One. 2017 Apr 20;12(4):e0176180. doi: 10.1371/journal.pone.0176180 (PMC5398710; doi:10.1371/journal.pone.0176180)
Supplement: S3 Table — Model 1: Adjusted for age, sex, race/ethnicity, height, weight, education, CT scanner manufacturer and milliamperes. Model 2: Additionally adjusted for total cholesterol, high-density lipoprotein cholesterol, triglycerides, hypertension, systolic blood pressure, diabetes, fasting glucose, creatinine, diuretic use, percent predicted FEV1 and percent emphysema, as well as current smoking status and pack-years for ever-smokers. *Cardiac output available for 1152 ever-smokers and 1018 never-smokers. (PDF) [file pone.0176180.s007.pdf]

	Ever-smokers (N=1226) Estimate (95% CI)	P-value	Never-smokers (N=1077) Estimate (95% CI)	P-value
**LV end-diastolic volume, mL**				
Model 1	-4.83 (-6.34, -3.31)	<0.001	-0.52 (-1.96, 0.92)	0.48
Model 2	-3.71 (-5.24, -2.18)	<0.001	0.18 (-1.27, 1.63)	0.81
**Stroke volume, mL**				
Model 1	-3.00 (-3.92, -2.09)	<0.001	-0.52 (-1.47, 0.42)	0.28
Model 2	-2.28 (-3.20, -1.35)	<0.001	-0.09 (-1.04, 0.85)	0.85
**Cardiac output, L/min***				
Model 1	-0.19 (-0.25, -0.12)	<0.001	-0.08 (-0.15, -0.01)	0.02
Model 2	-0.15 (-0.22, -0.09)	<0.001	-0.07 (-0.14, 0.002)	0.06
**LV mass, g**				
Model 1	-1.44 (-2.76, -0.13)	0.03	0.75 (-0.52, 2.01)	0.25
Model 2	-1.29 (-2.58, 0.004)	0.05	0.94 (-0.31, 2.18)	0.14
**LV mass/end-diastolic volume ratio, g/mL**				
Model 1	0.034 (0.021, 0.047)	<0.001	0.014 (0.002, 0.026)	0.02
Model 2	0.023 (0.010, 0.036)	<0.001	0.009 (-0.003, 0.022)	0.14
**LV ejection fraction, %**				
Model 1	-0.21 (-0.62, 0.19)	0.30	-0.03 (-0.47, 0.40)	0.88
Model 2	-0.18 (-0.60, 0.24)	0.40	-0.02 (-0.46, 0.42)	0.93
