# Supplementary material for: Pulmonary vascular volume, impaired left ventricular filling and dyspnea: The MESA Lung Study
Source: PLoS One. 2017 Apr 20;12(4):e0176180. doi: 10.1371/journal.pone.0176180 (PMC5398710; doi:10.1371/journal.pone.0176180)
Supplement: S4 Table — Model 1: Adjusted for age, sex, race/ethnicity, height, weight, education, CT scanner manufacturer and milliamperes. Model 2: Additionally adjusted for total cholesterol, high density lipoprotein cholesterol, triglycerides, hypertension, systolic blood pressure, diabetes, fasting glucose, creatinine, diuretic use, percent predicted FEV1 and percent emphysema, as well as current smoking status and pack-years for ever-smokers. *T1 time and extracellular volume fraction models also adjusted for heart rate and left ventricular end-diastolic mass. (PDF) [file pone.0176180.s008.pdf]

	Ever-smokers Estimate (95% CI)	P-value	Never-smokers Estimate (95% CI)	P-value
**Left atrial volume, mL**	N=1056		N=943	
Model 1	-1.31 (-3.08, 0.47)	0.15	-0.97 (-3.06, 1.13)	0.37
Model 2	-4.02 (-5.90, -2.14)	<0.001	-2.14 (-4.48, 0.19)	0.07
**Peak early diastolic strain rate, %/msec**	N=997		N=1055	
Model 1	0.001 (-0.004, 0.006)	0.74	-0.004 (-0.010, 0.002)	0.18
Model 2	-0.001 (-0.006, 0.005)	0.80	-0.004 (-0.011, 0.002)	0.17
**Strain relaxation index, msec/%**	N=926		N=1000	
Model 1	-0.04 (-0.10, 0.03)	0.27	0.06 (-0.01, 0.13)	0.11
Model 2	-0.01 (-0.08, 0.06)	0.87	0.06 (-0.02, 0.14)	0.15
**25-min post-contrast myocardial T_1 time, msec***	N=547		N=419	
Model 1	-6.08 (-10.22, -1.95)	0.004	-3.80 (-9.33, 1.72)	0.18
Model 2	-7.51 (-11.95, -3.07)	<0.001	-3.46 (-9.53, 2.61)	0.26
**Extracellular volume fraction, %***	N=237		N=169	
Model 1	0.49 (-0.02, 0.99)	0.056	0.45 (-0.20, 1.11)	0.17
Model 2	0.62 (0.08, 1.16)	0.026	0.58 (-0.18, 1.34)	0.14
