# Supplementary material for: Pulmonary vascular volume, impaired left ventricular filling and dyspnea: The MESA Lung Study
Source: PLoS One. 2017 Apr 20;12(4):e0176180. doi: 10.1371/journal.pone.0176180 (PMC5398710; doi:10.1371/journal.pone.0176180)
Supplement: S5 Table — Data are presented as % or mean±SD, except as noted. Abbreviations: HDL, high-density lipoprotein; COPD, chronic obstructive pulmonary disease; GOLD, Global initiative for chronic obstructive lung disease; IQR, interquartile range. *Calculated as pulmonary microvascular blood volume (cm3 blood/100 cm3 lung) x total lung volume on CT scans obtained at functional residual capacity, in 91 subjects. (PDF) [file pone.0176180.s009.pdf]

	All participants (N=142)
Age, years	67.9±7.0
Male, no. (%)	84 (59.2)
Race/ethnicity, no. (%)	
White	83 (58.5)
African-American	29 (20.4)
Hispanic	26 (18.3)
Chinese-American	4 (2.8)
Educational attainment, no. (%)	
High school degree or less	33 (23.2)
Some college	35 (24.7)
College degree	74 (52.1)
Body mass index, kg/m^2	27.4 (4.8)
Cigarette smoking status, no. (%)	
Former smoker	91 (64.1)
Current smoker	51 (35.9)
Pack-years	37.7±21.1
Hypertension, no. (%)	77 (54.2)
Systolic blood pressure, mmHg	121.3±15.2
Total cholesterol, mmol/L	4.8±1.0
HDL cholesterol, mmol/L	1.50±0.5
Triglycerides, mmol/L	1.23±0.6
Diabetes, no. (%)	25 (17.6)
Fasting glucose, mmol/L	5.8±1.0
Diuretic use, no. (%)	20 (14.1)
COPD, no. (%)	80 (56.3)
GOLD severity, no. (%)	
Mild	30 (21.1)
Moderate	37 (26.1)
Severe/very severe	13 (9.2)
Percent emphysema, median (IQR)	1.52 (0.72, 4.06)
Pulmonary microvascular blood volume, cm^3 blood/100 cm^3 lung	4.36±2.60
Total pulmonary microvascular blood volume, cm^3 blood*	141.9±77.2
LV end-diastolic volume index, mL	62.4±12.8
Stroke volume index, mL	37.7±7.7
Cardiac output, L/min	2.6±0.5
LV mass index, g	69.7±14.0
LV mass/end-diastolic volume ratio, g/mL	1.14±0.25
LV ejection fraction, %	60.7±7.3
Left atrial volume index, mL	33.7±10.2
Pulmonary vein cross-sectional area, cm^2	5.91±1.58
