# Supplementary material for: Pulmonary vascular volume, impaired left ventricular filling and dyspnea: The MESA Lung Study
Source: PLoS One. 2017 Apr 20;12(4):e0176180. doi: 10.1371/journal.pone.0176180 (PMC5398710; doi:10.1371/journal.pone.0176180)
Supplement: S6 Table — Abbreviations: LV, left ventricular; EDV, end-diastolic volume. Model 1: Adjusted for age, sex, race/ethnicity, height, weight, education and cohort. Model 2: Additionally adjusted for smoking status, pack-years, total cholesterol, high density lipoprotein cholesterol, triglycerides, hypertension, systolic blood pressure, diabetes, fasting glucose, creatinine, diuretic use, percent predicted FEV1 and percent emphysema. Model 3: Additionally adjusted for respective right ventricular parameter. *N = 141, 1 participant with uninterpretable right ventricular measures on MRI. †N = 139, 3 participants without left atrial volume or pulmonary vein area measurement. ‡Adjusted for right ventricular end-diastolic volume. N = 138, 1 participant with uninterpretable right ventricular measures on MRI. (PDF) [file pone.0176180.s010.pdf]

	Estimate (95% CI)	P-value
**LV end-diastolic volume, mL**		
Model 1	-6.28 (-10.53, -2.04)	0.004
Model 2	-5.76 (-10.01, -1.51)	0.008
Model 3*	-3.46 (-6.61, -0.30)	0.03
**Stroke volume, mL**		
Model 1	-4.07 (-6.52, -1.61)	0.001
Model 2	-3.39 (-5.87, -0.91)	0.007
Model 3*	-3.00 (-5.12, -0.89)	0.005
**Cardiac output, L/min**†		
Model 1	0.11 (-0.06, 0.29)	0.20
Model 2	0.10 (-0.07, 0.28)	0.11
Model 3*	-0.02 (-0.16, 0.12)	0.73
**LV mass, g**		
Model 1	1.27 (-2.69, 5.23)	0.53
Model 2	1.12 (-2.67,4.90)	0.56
Model 3*	2.20 (-1.44, 5.84)	0.24
**LV mass/EDV ratio, g/mL**		
Model 1	0.08 (0.03, 0.12)	<0.001
Model 2	0.06 (0.03, 0.10)	0.001
Model 3*	0.07 (0.04, 0.11)	<0.001
**LV ejection fraction, %**		
Model 1	-0.38 (-1.56, 0.81)	0.53
Model 2	-0.19 (-1.34, 0.97)	0.75
Model 3*	-0.31 (-1.38, 0.76)	0.57
**Left atrial volume, mL**		
Model 1†	-2.72 (-6.32, 0.88)	0.14
Model 2†	-1.79 (-5.59, 2.01)	0.36
Model 3‡	-1.32 (-4.96, 2.31)	0.39
**Pulmonary vein area, cm^2**		
Model 1†	-0.45 (-0.72, -0.18)	0.001
Model 2†	-0.41 (-0.69, -0.14)	0.003
Model 3‡	-0.36 (-0.62, -0.09)	0.009
